# Effect of self-determination theory-based integrated creative art (SDTICA) program on older adults with mild cognitive impairment in nursing homes: Study protocol for a cluster randomised controlled trial

**DOI:** 10.1186/s12877-023-03896-0

**Published:** 2023-04-20

**Authors:** Chen-shan Huang, Yuan-jiao Yan, Rong Lin, Wen-qian Sun, Yu Ye, Na-fang Wang, Hong Li

**Affiliations:** 1 The School of Nursing, Fujian Medical University, No. 1 Xueyuan Road, Shangjie Town, Fuzhou City, Fujian Province 350122 China; 2 Shengli Clinical Medical College, Fujian Provincial Hospital & Fujian Medical University, No. 134 Dongjie Street, Gulou District, Fuzhou City, Fujian Province 350001 China

**Keywords:** Art, Cluster-randomised controlled trial, Non-pharmacological intervention, Nursing home, Older adults, Self-determination theory

## Abstract

**Background:**

The cognitive benefits of early non-pharmacological approaches have been demonstrated in older adults with mild cognitive impairment (MCI). However, older adults living in nursing homes have more severe cognitive impairment problems and lower initiative and compliance to participate in complex interventions. Hence, it important to investigate more attractive and sustainable methods to prevent or delay cognitive decline. The present study adopts the self-determination theory (SDT) as a theoretical framework to innovatively develop an integrated art-based intervention for older adults with MCI in nursing homes in China and aims to evaluate its effects on cognitive function, mental health, and other health-related outcomes.

**Methods:**

The study is a nursing home-based, cluster randomised controlled trial (RCT) that targets older adults (aged ≥ 60 years) with MCI in Fuzhou City, China. All nursing homes in the area covered by Fuzhou City are invited to participate. Eligible nursing homes are randomised to one of two groups: intervention group (receive a 14-week, 27-session intervention) and waitlist control group (receive the usual care). The SDT-based integrated creative art (SDTICA) program reasonably adopts the SDT as a theoretical framework to innovatively develop an integrated art-based intervention for older adults with MCI in nursing homes. The primary (global cognitive function and psychological indicator) and secondary (daily activity function, social function, and specific domains of cognitive function) outcomes will be measured at baseline, after the intervention, and during follow-up.

**Discussion:**

This study aims to evaluate the effects of SDTICA program on neuropsychological outcomes in older adults with MCI and provide scientific evidence for art-based non-pharmacologic interventions in nursing homes, which may reduce dementia risk in older adults with MCI.

**Trial registration:**

The trial was prospectively registered at the Chinese Clinical Trials Registry with the registration number ChiCTR2200061681 on 30 June 2022.

**Supplementary Information:**

The online version contains supplementary material available at 10.1186/s12877-023-03896-0.

## Background

According to the 2018 World Alzheimer’s Disease Report [1], there are more than 50 million people with dementia worldwide, and the annual social and economic burden caused by dementia reaches US $1 trillion. Currently, there are more than 15 million older adults with dementia in China, accounting for 25% of the global total. The social and economic burden of dementia accounts for 1.47% of the gross domestic product, which is higher than the global average [2, 3]. Nevertheless, due to the irreversible course of dementia and the limitations of treatment, early diagnosis and early intervention have become the focus of dementia prevention and treatment. Hence, the early diagnosis and preventive intervention of mild cognitive impairment (MCI), a potential precursor disease of dementia, has increasingly attracted the attention of researchers. MCI is a transitional stage between normal aging and mild dementia [4]. The incidence of MCI in adults aged 60 years and above in China is 15.54%; further, approximately 10-20% of MCI cases transform into dementia every year, and the risk of dementia in people with MCI is 10 times that of healthy older adults [2, 5]. Therefore, promoting dementia prevention and MCI treatment and taking targeted intervention measures as soon as possible is expected to prevent or delay the occurrence and progression of dementia; dementia prevention has positive significance for promoting healthy aging.

Notably, institutionalisation can also negatively impact an individual’s global cognitive function. Due to the miniaturisation of family structure and the change of people’s concept of pension, China’s pension model has gradually changed from traditional family pension to institutional pension, and the number of older adults in nursing homes has gradually increased [6]. A 22-year follow-up study found that older adults in nursing homes showed greater cognitive decline than those who remained in the community [7]. The results of several studies suggest that the incidence of cognitive dysfunction in older adults in nursing homes in China is higher than the overall incidence in the country [6–8]. A possible reason of institutional cognitive decline may be a lack of cognitively stimulating activity, and studies have shown that people who are chronically exposed to cognitively stimulating environments maintain higher cognitive function with age [7, 9–11]. Although activities such as playing cards or watching television are common in nursing homes, several studies have shown that these leisure activities are not sufficient to maintain cognitive health [12–14]. Cognitive stimulation (CS) is a kind of non-pharmacological intervention, which refers to a series of non-specific cognitive intervention activities that stimulate thinking, attention, and memory in groups of people in a social environment; these activities include reality-oriented activities, art activities, and topic discussions to improve the patient’s cognitive or social function [15]. A Bayesian network meta-analysis of 13 randomised controlled trials (RCTs) suggested that CS may be the best intervention for improving cognitive function in patients with MCI [16]. Thus, it is urgent to pay close attention to the cognitive function of older adults with MCI in nursing homes, and to construct and implement preventive intervention programs cantered on CS, so as to delay cognitive decline, promote physical and mental health, and improve quality of life. However, the number of studies on older adults in nursing homes is limited, and there is a lack of intervention approaches suitable for older adults with MCI in nursing homes.

In recent years, the benefits of art-based CS for cognitive and emotional states of the older adults with cognitive impairment have been continuously verified [17]. Art therapy (AT) is defined as “a form of psychotherapy that uses art media as its primary mode of communication”, and is subdivided into categories such as visual arts, music, dance, drama, and literature [18]. This has been developed as an effective intervention strategy, as it provides older adults with a complex and rich environment by producing multisensory stimulation, challenging cognitive tasks, and increased social interaction [19]. A meta-analysis of 11 RCTs reported that AT can effectively stimulate and improve cognitive function and reduce depression in older adults by maximising the integration of socialization, work creation, and story sharing [20]; another meta-analysis also confirmed the positive effects of AT on cognitive function in older adults with MCI [17]. Considering the practical application of non-pharmacological interventions in older adults with MCI in nursing homes, AT may be a more culturally and contextually appropriate option. This is because the older adults with MCI in nursing homes generally have the characteristics of advanced age, poor mobility, and limited social interaction [7, 21, 22], which increase the requirements on the content elements, intervention modes, and difficulty settings of interventions. In contrast, differences in individual educational backgrounds, interest tendencies, thinking habits, and receptive abilities also increase the challenge of applying cognitive interventions to promote brain health in this group of people. AT that relies on creativity and nonverbal expression may be a better option. First, AT focuses on the integration of personal and internal experience in the whole process of creation, rather than the analysis and judgment of works of art [23]. Hence, participants who accept AT do not need to have any experience or background in artistic creation, and thus it has good applicability and acceptability for older adults with MCI in nursing homes. Second, AT provides older adults with MCI in nursing homes with a communication channel for non-verbal communication, creating more opportunities for social and interpersonal interaction, enabling them to freely express their inner feelings in a supportive environment and relieving anxiety, depression, and social isolation, while gaining new skills and a sense of well-being [24–26]. Kim et al. found that group AT played a significant role in improving depressive state and self-expression in patients with MCI [25]. In addition, Pérez et al. reported that group music therapy can effectively improve the cognitive function and emotional state of older adults with cognitive impairment in nursing homes [26]. Nevertheless, there is no agreement on which type of AT provides the most benefit for treating dementia [27]. A meta-analysis found that different types of AT (including visual arts, music, drama, and poetry) showed significant improvements in at least one outcome measure such as cognitive function, behavioural symptoms, and emotional state [27]. This suggests that combining different types of integrated AT may potentially show more cognitive benefits than using a single type of AT [28]. But most of the studies on the application of AT to older adults with MCI in nursing homes have great heterogeneity in terms of research design, diagnostic criteria, and intervention modes moreover, these studies are mainly small-sample and single-centre studies, with few follow-up evaluations to determine the long-term efficacy of the intervention. Overall, research evidence is of low quality and the efficacy of the intervention is unclear [17]. Additionally, the lack of motivational theoretical guidance in the construction and implementation of most intervention programs often leads to insufficient motivation to participate in interventions and decreased compliance in the older adults with MCI, which has become the primary factor hindering the promotion of these intervention programs in the social environment [29, 30].

There is robust evidence that interventions guided by motivation theory can have a significant effect on the maintenance of individual behavioural changes [31, 32]. Further, self-determination theory (SDT) is a motivational theory commonly used in behavioural interventions [33]. It emphasises improving autonomy support, which is consistent with the autonomous supportive environment provided by AT [34]. Motivation, which is the initiating factor of individual activities and an important factor in maintaining the continuity of activities, is considered to be the most powerful and stable predictor of activity at the individual level [35]. Meeting the three basic psychological needs of autonomy, competence, and relatedness, and then promoting the generation of internal motivation and the internalisation of external motivation is important for the promotion and maintenance of positive behaviour change in older adults, thereby helping individuals maintain behavioural changes for up to 12 months [36–39].Therefore, this study attempts to apply SDT in the design and implementation of intervention programs for the older adults with MCI in nursing homes, in order to more effectively trigger and maintain participants’ positive behavioural changes.

To these ends, we can reasonably adopt the SDT as a theoretical framework to innovatively develop an integrated art-based intervention for older adults with MCI in nursing homes in China and conduct a methodologically rigorous RCT; moreover, a 6-month follow-up will be conducted to evaluate its short- and long-term effects on neuropsychological outcomes in older adults with MCI. We hypothesized that the 14-week, 27-session SDT-based integrated creative art (SDTICA) program could serve as a non-pharmaceutical intervention to effectively improve cognitive, psychological, and social functioning in older adults with MCI in nursing homes.

## Methods/Design

### Study design, setting, and ethics

**Fig. 1:**
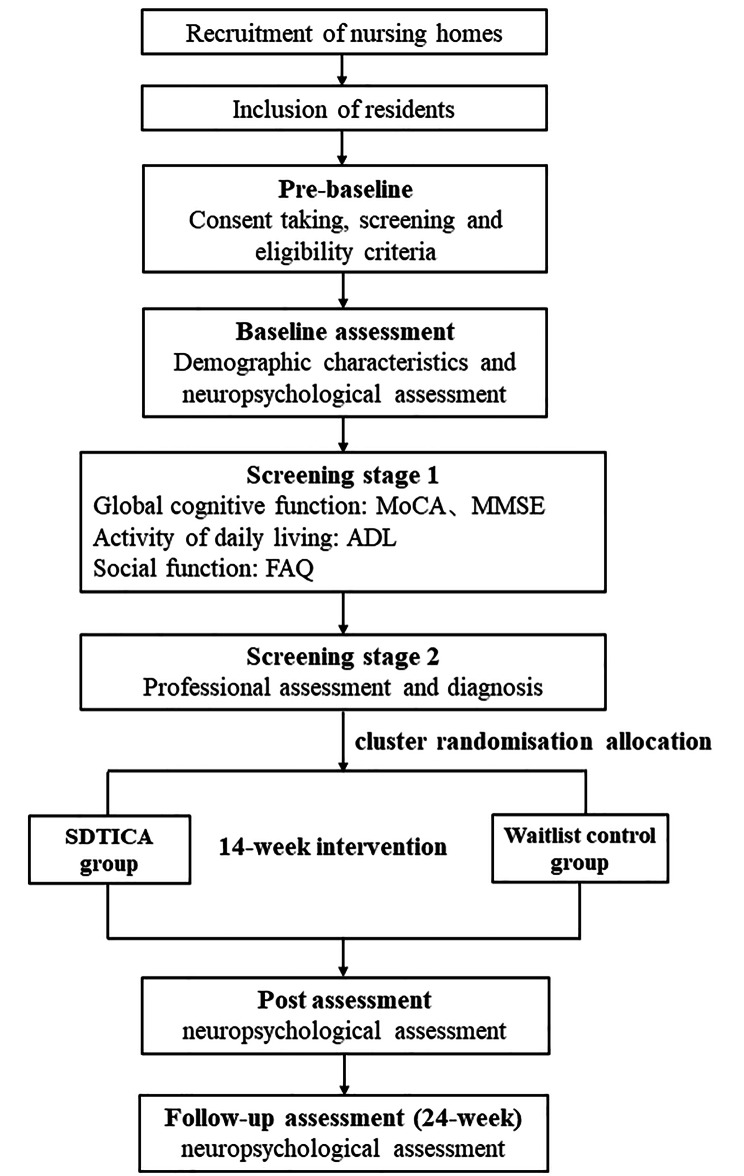
Flow diagram of the study design

The study is a cluster randomised, controlled, single-blind, parallel-group, multicentre trial conducted on older adults with MCI to assess the effects of the SDTICA program on cognitive function, mental health, and other health-related outcomes (Fig. 1). It will be conducted in nursing homes in Fuzhou City, China.

The nursing homes will be randomised on a 1:1 basis to the intervention (SDTICA program) or waitlist control (usual care) group. The waitlist control group will receive the usual care provided by the nursing home, and the intervention group will be exposed to the SDTICA program by professionally trained and qualified intervenors. The SDTICA program design will follow the Medical Research Council (MRC) guidelines on the evaluation and development of complex interventions [40]. A pilot trial will be conducted to assess the acceptability, safety, and feasibility of the SDTICA program. This study will be conducted, analysed, and reported in accordance with the Consolidation Standards of Reporting Trials (CONSORT) statement for cluster RCTs [40]. All procedures will be conducted in accordance with the Declaration of Helsinki. Ethics approval has been granted by the Ethics Committee of Fujian Provincial Hospital (K2022-05-015) and was registered in the Chinese Clinical Trials Registry with registration number ChiCTR2200061681 (trial registration data available at: http://www.chictr.org.cn/edit.aspx?pid=166566&htm=4).

### Nursing home recruitment and screening

All five-star nursing homes in Fuzhou City will be invited to participate in the study. Individuals recruited from nursing homes tend to have more advanced cognitive impairment compared with community-dwelling individuals [7]. The severity of cognitive impairment in study participants could impact their motivation and ability to participate in and adhere to a complex intervention [41, 42]. Hence, we will adopt multiple effective recruitment strategies. First, we will contact qualified nursing homes via telephone calls to explain the study. If a facility is interested in the study, we will visit the facility to provide more detailed information. Thereafter, prefeasibility assessment will be conducted to evaluate if nursing homes have resources to support recruitment and intervention, such as availability of social and care workers to assist in the study as well as a suitable venue for the SDTICA program. If the nursing home is eligible for selection and agrees to participate in the study, preselection screening will be conducted to identify eligible participants.

Nursing homes with the following criteria will be considered eligible:

1. Having a permit for the establishment of old-age care service institutions.
2. Rated as five-star level according to the Pension Institutions Grade Division and Assessment [43]; the rating is performed by professionals who are qualified in terms of grade assessment and training. The level (one- to five-star level) is rated based on a comprehensive consideration of the environment, facilities and equipment, operation management, and service provision of pension institutions. The higher the level, the stronger the comprehensive ability of pension institutions in the abovementioned four aspects.
3. Equipped with self-care area.
4. Agree to participate in this study and are able to provide venue and personnel support.

Nursing homes with the following criteria will be excluded:

1. Participating, has recently participated, or plans to participate in another trial that conflicts with the SDTICA program or data collection during trial participation.
2. Insufficient attention to this study, serious progress lag, and ineffective communication.
3. The presence of certain difficulties in patient recruitment or organization to perform intervention activities, resulting in failure to promptly and accurately complete the study plan, and if these difficulties cannot be solved after communication efforts.

### Participant recruitment and screening

Participants will be nursing home-dwelling older adults with MCI (aged at least 60 years). Participant recruitment will be performed through public advertisements (e.g., posters, leaflets, and brochures), media outreach (e.g., local newspapers and website-published feature stories about the potential benefits of art-based intervention for cognitive function among older adults with MCI), mobilization meetings (to introduce potential participants to the symptoms and harms of MCI, as well as the content and benefits of the SDTICA program), art-based experience activities, and word-of-mouth by the residents and social workers who will be mobilized to invite participants for screening. Potential participants who respond to the recruitment strategies will be screened for study eligibility in a two-stage process.

#### Screening stage 1 (SS1)

The purpose and process of the study will be fully explained to each participant, and a written informed consent form will be signed by consenting participants. Neurophysiological assessment will be performed by qualified researchers to evaluate participants’ global cognitive function using a combination of the Montreal cognitive assessment (MoCA) and Mini-Mental State Examination (MMSE) [44, 45]. In addition, the activity of daily living (ADL) and functional activities questionnaire (FAQ) will be used to assess daily and social competency [46, 47]. Participants who meet all the following criteria will proceed to the second screening stage: (1) MoCA score ≤ 13/14 for illiterate individuals, ≤ 19/20 for individuals with 1 to 6 years of education, or ≤ 24/25 for individuals with 7 or more years of education [48]; and (2) MMSE score = 24–30 [49]. Cutoff MoCA scores will be used to determine the presence of cognitive impairment, whereas MMSE scores will be used to confirm the absence of dementia [50].

#### Screening stage 2 (SS2)

SS2 will be performed to determine if cognitive impairment is sufficient to be diagnosed as MCI; SS2 will be performed within 1 week from SS1. A professional team of neurologists and nurses will assess participants’ cognitive function subdomains in detail, including memory, executive function, language, visuospatial abilities, attention, and reaction speed.

#### Diagnostic criteria

MCI diagnosis is based on Petersen criteria are as follows [51]: (1) memory complaint, preferably corroborated by an informant; (2) objective memory impairment for age (MoCA score ≤ 13/14 for illiterate individuals, ≤ 19/20 for individuals with 1 to 6 years of education, or ≤ 24/25 for individuals with 7 or more years of education); (3) essentially intact ADL (ADL score < 25 for age ≥ 75 years and < 23 for age < 75 years) [52]; and (4) not demented (MMSE = 24–30). The final clinical diagnosis will be made by experienced neurologists according to the Diagnostic and Statistical Manual of Mental Disorders, Fifth Edition [53].

#### Inclusion criteria

Eligible participants will meet the following criteria: (1) age ≥ 60 years, (2) diagnosis of MCI, (3) visual and auditory acuity adequate for neuropsychological testing, and (4) able to provide written informed consent and willingness to comply with study visits/testing over the intervention and assessment period.

#### Exclusion criteria

The exclusion criteria of participants are as follows: (1) severe physical dysfunction such as hearing and visual impairment that limits testing or SDTICA program application, (2) history of mental illness or congenital mental retardation, (3) coma, serious disease, or terminal-stage disease, (4) the presence of other neurological diseases that can cause brain dysfunction, (5) intake of any cognitive-impairing/-enhancing medications, and (6) those undergoing other non-pharmacological interventions.

### Sample size determination

The sample size was calculated based on MoCA score improvement reported in our previous study that evaluated the effect of creative expressive therapy on improving cognitive function in patients with MCI [54]. The MoCA scores were 24.68 ± 1.84 (intervention group) and 23.13 ± 1.68 (control group) in our previous study. Using PASS Version 11.0 software (NCSS, Kays-vile, Utah, USA), a sample size of 25 participants per group was determined as being sufficient to detect an effect with a type 1 error rate of 5% (α = 0.05) and 90% power (β = 0.10). To account for clustering, the sample size was calculated according to the requirements of the cluster RCT [55]. Given that interclass correlation coefficient = 0.01 [56], N (individual RCT the required sample size) = 50, “n” means sample size for each cluster, and “k” means number of clusters, the required sample size for each group was calculated. Considering a 15% attrition rate, a total of [(49.5*k)/(0.85*k-0.425)] participants are required.

### Randomisation, blinding, and allocation concealment

Cluster randomisation will be used to ensure blinding and avoid contamination between groups [40]. Randomisation will occur after participant recruitment, informed consent, and complete baseline assessment. A nursing home is referred to as one cluster in this study. We will randomly select eligible nursing homes from all five-star nursing homes in order to minimize the baseline differences in environment, facilities and equipment, operation management, and service provision. A researcher (not involved in participant recruitment and evaluation) will perform the randomisation of nursing homes via lottery. Allocation will be concealed from the assessors.

### Intervention

#### Intervention group

The SDTICA program was developed by our research team in consultation with art therapists, neurology experts, clinical nurse specialists, and social workers. The SDTICA program will consist of a 27-session syllabus of 45–60 min per session. A group approach of 8–10 people per group will be used [57]. If experienced facilitators are available, we will consider increasing the number of participants. The program will focus not only on cognitive enhancement, but also on psychological health and social interaction as well as engagement in meaningful leisure activities. The SDTICA program will be conducted in a nursing home activity room, and will be led by two experienced and qualified instructors who have been trained in AT and have certified proficiency and experience in teaching art methods to older adults. Furthermore, the nursing home will provide two more social workers (to assist in guiding activities) and a care worker (to provide medical security) to assist with the program. Program implementation will take into account participant engagement and study feasibility. According to MRC guidance for the development and evaluation of complex interventions, the program will be pretested in terms of feasibility via a pilot study [58]. The protocol of the SDTICA program is detailed in the Additional file.

There is evidence [59] that the activity theme integrating traditional cultural elements with Chinese characteristics and nostalgic elements is more suitable for the psychological characteristics of older adults in China and effectively stimulates the creative enthusiasm and interest of participants. Therefore, the program will combine the historical background experienced by older adults; integrate the elements of traditional Chinese culture and nostalgia; and cover the four theme types of “traditional festivals, historical nostalgia, exploration of imagination, and general themes”. Each session, which is structured and pre-planned during the study, will be divided into four phases: Phase 1 involves 5–10 min for introduction and warm-up activities, which are made up of thematic-related art materials exploring body warming or music games. Phase 2 involves 5 min for topic discussion. Instructions will be provided with written steps/slides showing the task sequence. During a conversation, participants will be questioned on a shared theme. Importantly, thematic exploration and discussions will build on the participants’ existing experience or foundation. Phase 3 involves 30 min for art production. In the form of peer support, participants will be asked to draw or create anything they consider relevant to the theme. Phase 4 involves 10 min for sharing, summary, and feedback. Participants will be encouraged to show and briefly introduce their own works within the scope of the group, and voluntarily share their feelings and experiences. The instructor will summarise the activity according to the participants’ feedback on the activity. In addition, regular art work exhibitions will be held to to recognise the efforts of the participants (e.g., art works will be pasted on art walls or placed on display cases). Additionally, possible adverse events including related to adherence to the study and participants’ attrition will be tracked and closely monitored by the instructor and other staff involved in the intervention.

Based on SDT, this program will be divided into three structural stages: (1) prophase, which focuses on relatedness and involves setting up a support group and using pair work between peers to help participants integrate into the group and be familiar with a wide variety of art materials; (2) metaphase, which focuses on competence and involves providing clear instructions/tips and guidance (e.g., through video, audio, and pictures related to the theme), inspiring participants to think and discuss, and guiding participants to explore various types of activities in order to gain relevant abilities; and (3) anaphase, which focuses on autonomy and involves providing creative art types and art materials for participants to choose from in the same event and encouraging them to create freely under specific themes in order to carry over creative abilities beyond the intervention. Before the intervention, we will hold health knowledge lectures and distribute MCI prevention knowledge handbooks to explain why older adults with MCI need to participate in the program. After the intervention, we will conduct the focus group to explore perceived benefits and personal barriers of participants and give them positive feedback regarding the intervention. In addition, we will combine session requirements, autonomy support strategies [60], and the behaviour change techniques by Michie et al. [61] to develop SDT-based behaviour change techniques (focus on relatedness, competence, and autonomy) suitable for this study; this will help promote participants’ autonomy motivation and adherence to intervention. The SDT-based behaviour change techniques are detailed in the Additional file.

#### Waitlist control group

Participants in the control group will engage in usual care delivered within the nursing home for 14 weeks after the baseline assessment. If the SDTICA program improves cognitive function and psychological indicators after 14 weeks, it will be applied after obtaining the consent of participants in the waitlist control group as compensation for their participation. Moreover, it can reduce the influence of participant expectation on intervention effect.

### Outcome measures

To explore the effects and mechanisms of the SDTICA program, various measurements will be obtained at baseline and immediately after the intervention. Outcome measures mainly include neuropsychological assessments. All outcome measures and assessment time points are shown in Table 1.

**Table 1:** SDTICA study outcome measures

Measures/Time point	Screening	Baseline	Post-intervention	Follow-up
		0 weeks	14 weeks	24 weeks
Eligibility assessment				
Diagnostic criteria	×			
Inclusion criteria	×			
Exclusion criteria	×			
**Informed consent**	×			
**Demographic characteristics**		×		
**Neuropsychological assessment**				
**Primary outcomes**				
General cognitive function (MoCA, MMSE)		×	×	×
Psychological indicators (GDS, SAS, MUNSH, GNSS, and QoL-AD)		×	×	×
**Secondary outcomes**				
Verbal memory (AVLT)		×	×	×
Language (CVFT and BNT)		×	×	×
Executive function (DST and STT)		×	×	×
Visuospatial skill (CDT)		×	×	×
Social function (FAQ and LSNS)		×	×	×
Physiological function (ADL)		×	×	×
**Qualitative outcome evaluation**			×	×

×, Time point in the trial at which the assessment will take place. MoCA, Montreal cognitive assessment; MMSE, Mini-Mental State Examination; GDS, Geriatric Depression Scale; SAS, Self-Rating Anxiety Scale; MUNSH, Memorial University of Newfoundland Scale of Happiness; GNSS, General Needs Satisfied Scale; QoL-AD, Quality of life-Alzheimer’s disease; AVLT, Auditory Verbal Learning Test; CVFT, Category Verbal Fluency Test; BNT, Boston Naming Test; DST, Digit Span Test; STT, Shape Trail Test; CDT, Clock Drawing Test; FAQ, Functional Activities Questionnaire; LSNS, Lubben Social Network Scale; ADL, Activity of Daily Living

#### Demographic characteristic measurements

Demographic characteristics (e.g., age, sex, marital status, education level, and socioeconomic status), medical history, usage of drugs, and leisure activities will be collected at baseline.

#### Neuropsychological assessments

##### Primary outcome

The primary outcomes, which include global cognitive function and psychological indicator, will be measured using the MoCA, MMSE, Geriatric Depression Scale [62], Zung Self-Rating Anxiety Scale [63], General Needs Satisfied Scale [64], Memorial University of Newfoundland Scale of Happiness [65], and Quality of life-Alzheimer’s disease questionnaire [66].

##### Secondary outcomes

The secondary outcomes include daily activity function, social function, and specific domains of cognitive function. Specific domains of cognitive function were measured as follows: (1) memory using the Auditory Verbal Learning Test [67], (2) executive function using the Shape Trail Test [68], (3) language using the Category Verbal Fluency Test [69] and Boston Naming Test [70], (4) attention using the Digit Span Test [71], and (5) executive function using the Clock Drawing Test [72]. Daily activity function will be evaluated with ADL. Social function will be assessed using the Lubben Social Network Scale [73] and FAQ. The cited references provide more information on the sensitivity and specificity of the measurement tools.

### Data collection

Data collection for the outcome variables pertaining to participants with MCI will be performed in nursing homes by trained researchers. Data will be collected at three time points: baseline, after 14 weeks of intervention, and at 24 weeks of follow-up. To reduce the risk of measurement bias, outcome assessors and statistical analysts will be blinded to the intervention and control allocation and will not be involved in the implementation of the intervention. Data will be encoded independently by two research associates, and inconsistencies will be double checked with the data source to ensure data accuracy.

### Data analysis

The study will be analysed and reported according to the CONSORT statement for cluster RCTs. The data analyses will be performed using IBM SPSS Statistics Version 21.0 software (IBM, Armonk, New York, USA). Demographic and other baseline characteristics will be summarised using descriptive statistics. Continuous variables with normal distribution will be presented as mean ± standard deviation; non-normal variables will be reported as median (interquartile range). The balance of the baseline data of the intervention and control groups will be compared using the two independent samples t-test, Chi-square test, Fisher’s exact test, and Mann-Whitney U rank-sum test. A mixed-effects model accounting for clustering effects will be used to obtain effect estimates, 95% confidence intervals, and p-values for all outcomes; this will demonstrate whether objectively measured neuropsychological test scores improved in the intervention group compared with the control group at 14 and 36 weeks.

In addition, some potential confounders such as age, sex, and education level will be adjusted. To minimize selection bias, statistical analyses will be based on an intention-to-treat (ITT) approach [74], and missing data will be imputed using the multiple imputation method. As a sensitivity analysis, we will perform per-protocol analyses [75], including only the participants who completed the entire intervention process with compliance greater than 80% and outcome assessment at two time points. A value of *P* < 0.05 will be considered significant and the alpha level will be two-sided. Data management will be the primary responsibility of the research team. All data will be securely stored in an electronic database and accessible only to researchers who meet the research ethics approval.

## Discussion

The aim of this study is to evaluate the effects of the SDTICA program on neuropsychological outcomes in older adults with MCI. The efficacy of art-based activity as a non-pharmacological intervention has been demonstrated, especially in cognitive functioning [76, 77]. In addition, art-based activities generally have additional benefits in terms of improving emotional state, strengthening social interaction, and enhancing quality of life and well-being of older adults [76, 77]. However, due to the limitations and heterogeneity of existing study methods, the efficacy of AT remains controversial, and many individuals have poor motivation and compliance to participate in the intervention [17, 27, 29]. Several studies conducted on art-based activities involving diverse forms of artistic expressions showed that these activities had positive effects on the cognitive function and self-efficacy of older adults with MCI [76, 77]. This suggests that combining different forms of expressive art activities may be an effective approach to delay the deterioration of cognitive function. Thus, we designed the SDTICA program that integrates visual art, music, dance, and poetry, and innovatively implemented the program under the guidance of the SDT framework, in order to more effectively trigger and sustain the positive behaviour change of older adults with MCI. Furthermore, this program may be a low-cost model of intervention that can easily be integrated in the existing services for older adults with MCI in nursing homes. In addition, rigorous methodologic designs will be adopted to minimize bias, including cluster randomisation, blinding, control group use, phased recruitment strategy, stringent eligibility screening, and pilot trial. Clusters will be randomly assigned after recruiting study samples and seeking informed consent from residents. Further, potential selection bias in the control and intervention groups will be controlled using the abovementioned strategies. All collected data will be included in the ITT analysis.

## Limitation

There are several limitations in this study. First, at the individual level, there may be selection bias because individuals who agree to participate in the study tend to be more motivated. Second, due to the complex and multi-faceted nature of the SDTICA program, even if participants were briefed in advance on the research requirements and procedures, there may be biases in the implementation process that may deviate from the intended intervention. Lastly, due to objective factors such as personnel allocation and distance, the measurement time points for some results may be slightly different between the intervention and control groups. However, we will do our best to limit these differences.

## Conclusion

This study will provide insight into the efficacy of integrated art-based cognitive intervention on delaying cognitive decline in older adults with MCI and improving their mental health, quality of life, and well-being. If the implementation of the intervention is effective, the protocol will be made available to nursing homes that are interested in offering non-pharmacological interventions to older adults with MCI.

## Electronic supplementary material

Below is the link to the electronic supplementary material.

Supplementary Material 1

## Data Availability

Data can be obtained from the corresponding author upon reasonable request.

## Abbreviations

AT: Art therapy
ADL: Activities of daily living
CS: Cognitive stimulation
CONSORT: Consolidation Standards of Reporting Trials
FAQ: Functional activities questionnaire
ITT: Intention-to-treat
MCI: Mild cognitive impairment
MRC: Medical Research Council
MMSE: Mini-mental state examination
MoCA: Montreal cognitive assessment
RCT: Randomised controlled trial
SDT: Self-determination theory
SDTICA: Self-determination theory-based Integrated Creative Art
SS1: Screening stage 1
SS2: Screening stage 2
