# Supplementary material for: Effect of self-determination theory-based integrated creative art (SDTICA) program on older adults with mild cognitive impairment in nursing homes: Study protocol for a cluster randomised controlled trial
Source: BMC Geriatr. 2023 Apr 20;23:238. doi: 10.1186/s12877-023-03896-0 (PMC10120181; doi:10.1186/s12877-023-03896-0)
Supplement: Supplementary file 1 — Supplementary Material 1 [file 12877_2023_3896_MOESM1_ESM.docx]

**Additional file**

**Effect of Self-determination Theory-based Integrated Creative Art (SDTICA) Program on Older Adults with Mild Cognitive Impairment in Nursing Homes: Study Protocol for A Cluster Randomised Controlled Trial**

**Key Words:** Art; Cluster-randomised controlled trial; Non-pharmacological intervention; Nursing home; Older adults; Self-determination Theory

**Running Head:** Effects of SDTICA in Older Adults with MCI in Nursing Homes

**List of additional files**

**1. Protocol of SDTICA program sessions**

**2. SDT-based behaviour change techniques of the SDTICA program**

**1. Protocol of SDTICA program sessions**

| **Session** | **Domains of SDT** | **Theme** | **Form** |
| --- | --- | --- | --- |
| Session 1-9  (prophase) | Relatedness | You run after me | Doodle |
|  |  | Heart to heart | Rubbing painting |
|  |  | Spring Festival fireworks | Felt collage |
|  |  | Fingertip flowers | Gesture dance |
|  |  | Love | Paper quilling |
|  |  | The most beautiful trip | Clay painting |
|  |  | Solar term | Music, poetry |
|  |  | Plum blossom | Chinese ink painting |
|  |  | Leaf collage | Collage, painting |
| Session 10-18  (metaphase) | Competence | Animal world | Scratch painting |
|  |  | Valentine’s Day | Twist stick bouquet |
|  |  | Chinese clothing | Crepe paper handmade |
|  |  | The Army’s Day | Music, poetry |
|  |  | Autumn | Cotton swab painting |
|  |  | The scenery outside the window | Chinese watercolour painting |
|  |  | Ancient characters | Painting |
|  |  | Myths and legends | Crayon drawing |
|  |  | Dance to the poetry | Gesture dance, poetry |
| Session 19-27  (anaphase) | Autonomy | Food in my memory | Painting, music, dance, poetry, etc. |
|  |  | The Mid-Autumn festival | Painting, music, dance, poetry, etc. |
|  |  | My career | Painting, music, dance, poetry, etc. |
|  |  | My hometown | Painting, music, dance, poetry, etc. |
|  |  | My motherland | Painting, music, dance, poetry, etc. |
|  |  | It’s me | Painting, music, dance, poetry, etc. |
|  |  | Happy time | Painting, music, dance, poetry, etc. |
|  |  | Important life experiences | Painting, music, dance, poetry, etc. |
|  |  | Harvest time | Painting, music, dance, poetry, etc. |

**2. SDT-based behaviour change techniques of the SDTICA program**

| **Domains of SDT** | **Component** | **Behaviour change techniques** |
| --- | --- | --- |
| **Autonomy** | **Offering a clear rationale and explanation** | - Hold health knowledge lectures to explain why the program is important for participants before the intervention. - Supporting information will be provided by distributing printed materials such as health manuals and broadcasting videos. |
|  | **Provide support and reduce control** | - Use neutral language during interpersonal communication (e.g., “may” and “could”, and not “should” or “must”) and reduce stress and requirements when talking about disease information or activities. - Inform participants of the topic before the next session and hand out paper session schedule. |
|  | **Providing options** | - Activity type: 27 group sessions, which consist of visual arts, music, dance to move, poetry and so on. - Art material: Rich and user-friendly art materials, and the novelty of materials should not be beyond the ability of participants. |
|  | **Acknowledge individual opinions and feelings** | - Conducting focus group to explore perceived benefits and personal barriers. - Acknowledging and accepting expressions of negative affect of the participants. |
| **Competence** | **Prompts/cues** | - Provide clear instructions/tips and guidance and inspire participants to think. - Provide a demonstration or template to help participants to be familiar with the creation method. |
|  | **Behavioural practice** | - Sufficient practice time should be included in each session. |
|  | **Graded tasks** | - Focus on the diversity and differences of participants and consider their needs, abilities, and interests. Provide tasks with appropriate difficulty levels based on participant level of ability. |
|  | **Feedback on behaviour** | - Seek to understand the reason behind participants’ behaviours (e.g., Task may be too easy/difficult, or participants may encounter other obstacles) and address the issue in time. - Assign a facilitator to engage late/low interest/learning late participants in one-to-one activities or assist participants who need more attention so that the main activity can continue uninterrupted. - Provide feedback on performance of the activity behaviour and inform the participants how they can improve activity levels. |
| **Relatedness** | **Social support** | **Intervenor support:**   - Set up a support group and focus on participants’ feelings and meet their needs. - Encourage communication and self-expression and offer non-specific praise or rewards for behaviour. |
|  |  | **Peer support:**   - Use pair work to encourage those who complete the task first to help their peers. - Communication and sharing among peers. |
|  |  | **Family support:**   - Set up a WeChat group for participants and their families and publish session notifications. - Encourage participants to share art works with their families. |
